# Proteome Profiling of Diabetic Mellitus Patient Urine for Discovery of Biomarkers by Comprehensive MS-Based Proteomics

**DOI:** 10.3390/proteomes6010009

**Published:** 2018-02-06

**Authors:** Yoshitoshi Hirao, Suguru Saito, Hidehiko Fujinaka, Shigeru Miyazaki, Bo Xu, Ali F. Quadery, Amr Elguoshy, Keiko Yamamoto, Tadashi Yamamoto

**Affiliations:** 1 Biofluid Biomarker Center, Niigata University, Niigata 950-2181, Japan; hirao@ccr.niigata-u.ac.jp (Y.H.); scn.nth@gmail.com (S.S.); dext007@hotmail.com (H.F.); kyonami-bbc@ccr.niigata-u.ac.jp (B.X.); fquadery@yahoo.com (A.F.Q.); amr_biotech2006@yahoo.com (A.E.); yamamotok-bbc@ccr.niigata-u.ac.jp (K.Y.); 2 Department of Pediatrics, National Hospital Organization Niigata Hospital, Kashiwazaki 945-8585, Japan; 3 Department of Nephrology, Shinrakuen Hospital, Niigata 950-2087, Japan; ys5n-oosg@asahi-net.or.jp

**Keywords:** urine, biomarker, diabetic mellitus

## Abstract

Diabetic mellitus (DM) is a disease that affects glucose homeostasis and causes complications, such as diabetic nephropathy (DN). For early diagnosis of DN, microalbuminuria is currently one of the most frequently used biomarkers. However, more early diagnostic biomarkers are desired in addition to microalbuminuria. In this study, we performed comprehensive proteomics analysis of urine proteomes of diabetic mellitus patients without microalbuminuria and healthy volunteers to compare the protein profiles by mass spectrometry. With high confidence criteria, 942 proteins in healthy volunteer urine and 645 proteins in the DM patient urine were identified with label-free semi-quantitation, respectively. Gene ontology and pathway analysis were performed with the proteins, which were up- or down-regulated in the DM patient urine to elucidate significant changes in pathways. The discovery of a useful biomarker for early DN discovery is expected.

## 1. Introduction

Urine remains one of the preferable biological samples for discovery, diagnosis and disease monitoring, due to the non-invasive nature of its collection and to its simple matrix compared to other blood-derived fluids [1]. Urine collection is simple and non-invasive, and an available volume is relatively abundant compared to other biological fluids. It contains information from systemic to local tissues via extracellular vesicles, proteins and small molecules. However, despite its lower range of protein concentrations, highly abundant proteins such as albumin, representing 25% of the total protein amount, are still a hurdle to the complete characterization of the urinary proteome, as it may mask the less abundant proteins [2]. Differential protein profiles and abundances are often observed by proteomics in urine from patients with various diseases [3]. The proteome represents the entire profile of proteins that are expressed in a biological sample under a defined condition. Proteomics may provide a reliable vantage point for cellular stress responses and other changes. The standardized definition of a proteomics biomarker has been proposed to be: “a specific peptide or protein that is associated with a specific condition, such as the onset, manifestation, or progression of a disease or a response to treatment” [4].

Diabetic mellitus (DM) is a group of metabolic disorders, in which there are high blood glucose levels over a prolonged period. Symptoms of high blood glucose include frequent urination, increased thirst and hunger. Furthermore, DM can cause many complications such as diabetic nephropathy (DN), retinopathy and neuropathy. Serious long-term complications are: cardiovascular disease, stroke, chronic kidney disease, foot ulcers and damage of the eyes. As of 2015, an estimated 415 million people had diabetes worldwide, with the Type 2 DM making up about 90% of these cases. Type 2 DM cases are often complicated by persistent albuminuria (>30 mg/g creatinine/day), progressive reduction in glomerular filtration rate (GFR), and hypertension [5]. Although microalbuminuria (30–300 mg/g/day) remains the gold standard for early detection of DN, it is not a sufficiently accurate predictor of the risk due to some limitations [6,7]. Thus, early discovery of novel biomarkers for prediction of the DN risk is expected to prevent the occurrence of end-stage kidney disease. 

In this study, we compared urine proteomes between DM patients without microalbuminuria and healthy volunteers (HV) to find differences in the profile of the proteomes or to identify biomarkers for early discovery of DN.

## 2. Materials and Methods

### 2.1. Urine Samples

Urine samples were collected from healthy volunteers (*n* = 5) and DM patients without microalbuminuria (*n* = 5). After collection, urine was centrifuged at 1000 g for 15 min and the supernatant was transferred to a new collection tube to remove cell debris and then aliquoted in 1.5 mL tubes to store at −20 °C until use. Table 1 described that the cohort data of samples used in this study.

### 2.2. Urine Protein Preparation

The urine proteins were precipitated by Methanol/chloroform precipitation from 500 µL of urine. Frozen urines were thawed at 37 °C in a water bath at 37 °C for 10 min before precipitation. An equal volume of methanol and one quarter of the volume of chloroform were added to the urine sample, then it was mixed well for 5 min. The sample was centrifuged at 19,000 *g* at 25 °C for 15 min. The supernatant was discarded without interfering with the interface layer by pipette. Then, an equal volume of methanol was added to the sample again and it was mixed gently for 5 min. The sample was centrifuged at 19,000 *g* at 25 °C for 15 min. Finally, the supernatant was removed and the obtained proteins were dried by air.

### 2.3. Tryptic Peptide Preparation

Precipitated proteins were dissolved in 100 µL of 8 M urea/50 mM Tris-HCl (pH 8.0) buffer. The sample was treated by 1 µL of 1 M dithiothreitol at RT for 1 h and 8 µL of 500 mM iodoacetamide at RT for 1 h with shading. The alkylation was stopped by 1 µL of 1 M dithiothreitol and then diluted eight times by 50 mM Tris-HCl (pH 8.0). For the digestion, 1 µg of trypsin (Agilent, Santa Clara, CA, USA) was added to the sample and incubated at 37 °C for 16 h with shaking. The digestion was stopped by 50% of trifluoro acetic acid (TFA).

The digested sample was purified by C18 spin column (GL Science, Tokyo, Japan) according to the manual. Briefly, a C18 spin column was activated by 100% and 50% acetonitrile sequentially and then equilibrated by 0.2% formic acid with centrifuging at 3000 *g* for 30 s. After conditioning, the sample was loaded into the spin column and centrifuged at 3000 *g* for 90 s. Then, trapped peptides were washed with 0.2% TFA twice and eluted by 95% acetonitrile with 5% formic acid. The eluted sample was dried up by VEC-260 vacuum dryer (Iwaki, Tokyo, Japan). The sample was re-suspended by 0.1% formic acid and the peptide concentration was measured by Nano drop 1000 (Thermo Fisher Scientific, Bremen, Germany). The sample was stored at −80 °C until use.

### 2.4. LC-MS/MS Analysis and Protein Identification

Samples were analyzed on a QExactive plus (Thermo Fisher Scientific, Inc. Bremen, Germany) in data dependent acquisition. Peptides were resolved by nanoflow liquid chromatography system (nLC1000, Thermo Fisher Scientific, Inc.) on a trap column (2 cm × 75 µm Acclaim Pepmap 100 column) and a separation column (12.5 cm × 75 µm NTCC-360) at a 300 nL/min with a multistep gradient. Mobile phase A: water with 0.1% formic acid; mobile phase B: acetonitrile with 0.1% formic acid. A quantity of 500 ng of peptides were injected and eluted from the analytical column with in a linear gradient of 2%B to 35%B in 120 min. The mass spectrometer was obtained in positive ion mode in the scan range MS and MS/MS of 350–1800 *m*/*z* and 200–2000 *m*/*z*, respectively. The 15 most intense peaks with charge state 2 and 3 were selected from each survey scan and subjected to CID fragmentation. The MS and MS/MS scan parameter settings were as follows: collision energy, 35%; electrospray voltage, 2.0 kV; capillary temperature, 250 °C and isolation windows 4 m/z. All MS files (.raw) are accessible from the JPOST repository at URL: http://jpostdb.org/.

All MS and MS/MS data were analyzed by Proteome Discoverer TM (v2.1, Thermo) for protein and peptide identification with SEQUEST HT algorithms. The data were queried against a Uniprot/SWISS-prot database (v2015-08; Homo sapiens 20,203 sequences). All database searches were performed using a precursor mass tolerance of ± 10 ppm, fragment ion mass tolerance of ±0.02 Da, enzyme specificity was set to trypsin and maximum missed cleavages values of 2. Cysteine carbamidomethylation was set as fixed modification. A percolator was used to adjust the false discovery rate (FDR) to 1% on peptide level. The emPAI (exponentially modified protein abundance index) value of identified proteins was used for non-label quantification [8]. For the data treatment, we first extracted the Uniprot accession, which has emPAI value. Then, identified proteins were filtered by an expression level of more than three of five samples for strict filtration. The datasets were compared by Venny (BioinfoGP, http://bioinfogp.cnb.csic.es/tools/venny) in this study. Gene Ontology (GO) analysis and Lyoto Encyclopedia of Genes and Genomes (KEGG) pathway were analyzed by the Reactome [9]. The result of GO analysis was performed to draw the rader graph by microsoft EXCEL software. 

## 3. Results

**Figure 1:**
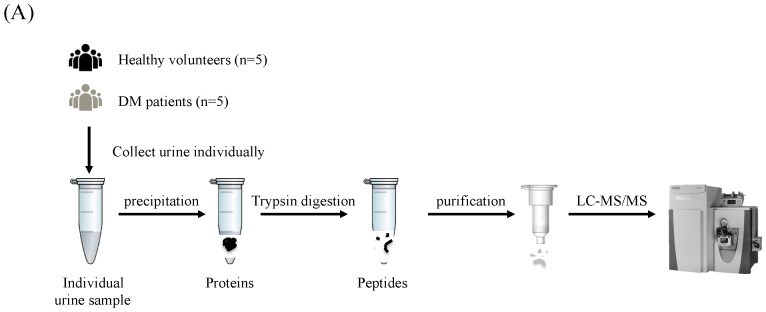

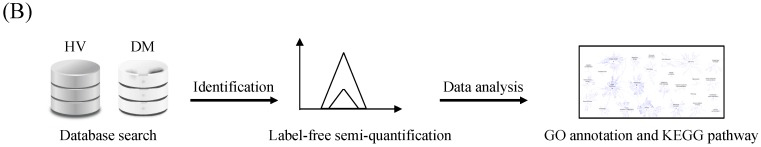
Schematic illustration of the urine proteomic screening process. (**A**) The workflow of MS analysis for proteomics approach. Collected urine samples were precipitated and digested by trypsin in solution. Purified peptides were injected into the MS spectrometry. (**B**) The data acquired by MS instrument were entered into to the database search for protein identification. Label-free semi-quantification was performed using by emPAI value and subjected to Gene Ontology (GO) annotation and KEGG pathway analysis.

To achieve a comprehensive analysis of the urinary proteins by LC-MS/MS, a duplicate analysis of the same sample was performed for urine samples from 5 healthy volunteers and 5 non-microalbuminuric DM patients (Figure 1). The amount of each protein was semi-quantitated by the emPAI value and compared statistically between the two groups. The proteins up- or down-regulated in the DM patient urine were analyzed by the GO annotation and KEGG pathway tools to elucidate the characteristics of the proteins and pathways.

### 3.1. Protein Identification and Label-Free Semi-Quantification

**Figure 2:**
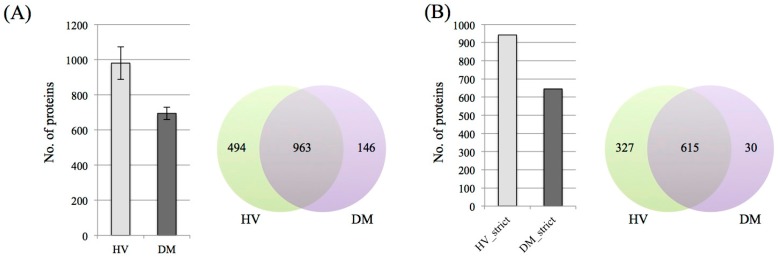

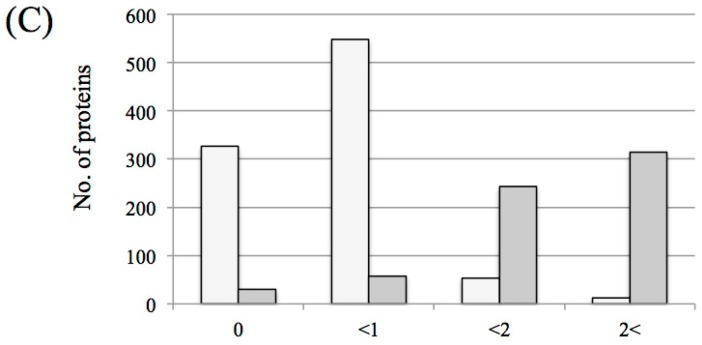
Protein identification and profile of each group. (**A**) Average non-redundant protein identification numbers from each group with <1% false discovery rate (FDR) at peptide level. The error bar indicated standard deviation. (**B**) Venn diagram of strict filtered protein group. (**C**) The distribution of up and down-regulated proteins between HV and DM in strict filtering. White and gray bar indicate “increase” and “decrease”, respectively.

The average numbers of proteins identified in the HV and DM patient urine were 979 and 694, respectively (Figure 2A). From the Venn diagram, 494 unique proteins of healthy subjects, 146 unique proteins for DM subjects and 963 shared proteins were demonstrated. Furthermore, proteins identified in more than three out of five samples were regarded as proteins with more strict identification (Figure 2B). From the result, the total number of identifications did not change greatly and unique and shared protein numbers were significantly decreased. To estimate the quantity of each protein, we employed the emPAI value for comparison and extracted highly changed proteins from strict protein identification. In this study, results that were more than 2 times greater were used for comparison between healthy and DM subjects. The number of proteins that increased in DM cases was only 13. On the other hand, 314 proteins were decreased. Interestingly, the rate of decrease was greatly shifted (Figure 2C).

### 3.2. Functional Enrichment Analysis

**Figure 3:**
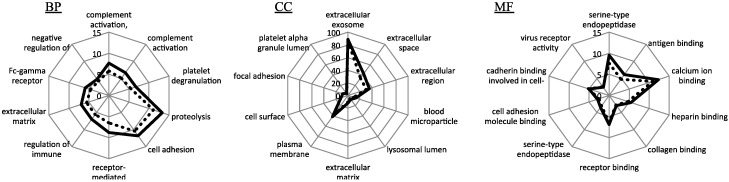
Rader graph showing the GO annotation analysis. From the left, Biological Process (BP), Cellular Component (CC) and Molecular Function (MF). HV and DM were indicated with blue and red lines, respectively.

To profile each healthy and DM dataset, the proteins which were identified were performed on DAVID (Figure 3). From the result, all the GO annotation, Biological Process (BP) and Cellular Component (CC) and Molecular Function (MF), distributed a similar profile in each healthy and DM group. Regarding the ratio of proteins in each GO annotation, the DM group showed a slightly high distribution. In GO CC analysis, 86.3% and 89% of proteins were related with an extracellular component in the healthy and DM groups, respectively.

### 3.3. Pathway Analysis

**Figure 4:**
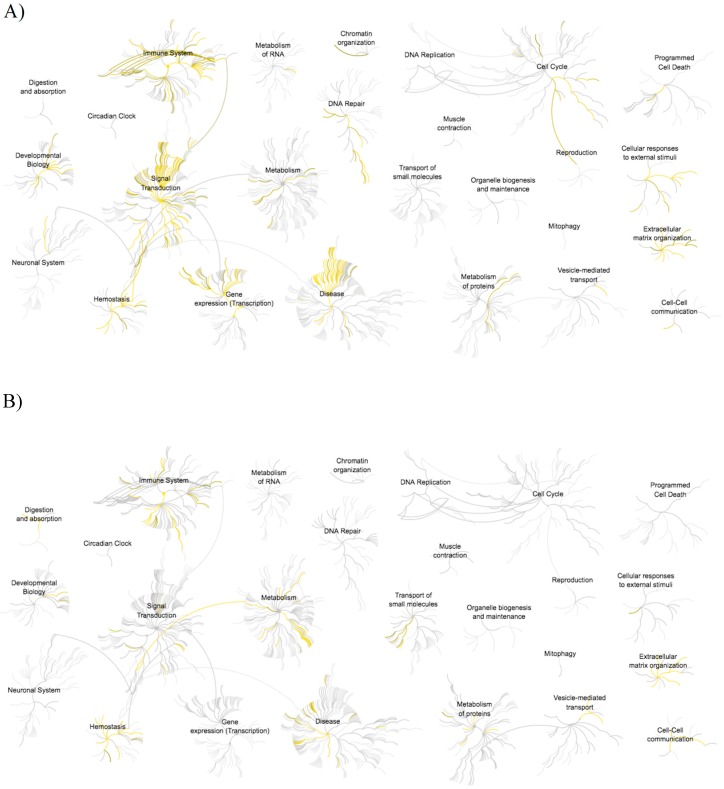
The result of pathway analysis by Reactome. (**A**) >2.0 increased and (**B**) <0.5 decreased in the diabetic mellitus (DM) patient.

Figure 4 shows the significantly enriched pathway of the up-regulated and down-regulated proteins in the DM group and the top 10 pathways were indicated in Table 2. From the reactome analysis, the Diseases of signal transduction, Signaling by Receptor Tyrosine Kinase, PI3K/AKT signaling in Cancer and Signal transduction pathway contributed to the up-regulated proteins group. On the other hand, the down-regulated proteins group indicated that several pathways were related to a high p-value. This can be seen, e.g., in the results for Neutrophil degranulation, Platelet degranulation, Regulation of IGF transport and uptake by IGFBPs, Response to elevated platelet cytosolic Ca^2+^ and Innate immune system. Furthermore, Metabolism, Homeostasis and Disease reaction were highly enhanced compared with the up-regulated proteins group in Reactome analysis. Finally, the down-regulated group of proteins related to Immune System and Signal Transduction pathways were highly decreased. 

## 4. Discussion

In recent years, clinical urinary proteomic analyses have been widely used to discover biomarkers for disease discovery, diagnosis and monitoring [1,10]. A comprehensive and representative proteome database of normal human urine is critically important as the background of a disease proteome for discovering biomarkers and the source of candidate proteins/peptides for targeted proteomics [1]. In the case of DM diagnosis, measurement of the blood glucose level has been widely used. Unfortunately, the progression of DM means that DN involves serious complications, and, except for microalbuminuria, biomarkers for DN diagnosis are not available in current clinical laboratory tests. However, microalbuminuria is an important biomaker in Type 2 DM, and is frequently used in population-based screenings [11,12,13]. Early diagnosis of DN is still difficult using only microalbuminuria and many cases of DN onset without microalbuminuria or proteinuria have been reported.

In this study, we profiled urine proteins, which identified in healthy volunteers and non-microalbuminuria DM patients. The numbers of proteins were 942 and 645 in HV and DM with strict identification, respectively. Even though the numbers may be not very high, they are comparable to previous ones and higher than a current report [14]. We could identify proteins as up-regulated in the DM patient urine. These have been partially reported as elevated proteins in Type 2 DM patient urine [15]. Focusing on down-regulated protein groups, the pathogenesis of DN involves the functional derangement and structural remodeling of the kidney, triggered by hyperglycemic injury, which are linked to changes in several cellular events and activation of signaling pathways [16]. Hence, the proteins related to protein-protein interaction, cell adhesion, cell-cell interaction at interendothelial junction and maintain were down-regulated. From our results, some of the proteins that were identified in the down-regulated group were related to protein-protein interaction or cell adhesion. The result of the pathway analysis also correlated to the DM patient and it was also linked to the down-regulated proteins group. Many immune system-related pathways were significantly different when compared with the up-regulated protein group.

## 5. Conclusions

With the wide application of the urinary proteome in clinical research, the construction of a representative and informative profile has become critically important. We performed a comprehensive label-free semi-quantification analysis of urine proteomes in healthy volunteers and DM patients to profile their difference. The profiles of each group were partially similar but distinctly different. In particular, the down-regulated protein group in the DM patient urine was remarkable. Our data facilitate further analysis of DM patient urine to discover novel and clinically useful biomarkers in the future.

## Figures and Tables

**Table 1:** List of the Top 10 pathways which are affected by the proteins (up- or down-regulated) for a DM patient. In total, 340 and 344 proteins were analyzed, respectively. ND, not done.

Group	Gender	*n*	Age	Alb/Cre	Proteinuria	eGFR	HbA1c
DM patient	male	5	68 ± 3	5.2 ± 3.1	-	2.2 ± 1	6.4 ± 0.7
HV	male	5	56 ± 4.4	ND	-	ND	ND

**Table 2:** List of the Top 10 pathways, which were affected by the up- or down-regulated proteins for the DM patient. In total, 340 and 344 proteins were analyzed, respectively.

Pathway (Increased)	Count	*p* Value
Diseases of signal transduction	50	1.11 × 10^−16^
Signaling by Receptor Tyrosine Kinase	53	1.11 × 10^−16^
PI3K/AKT Signaling in Cancer	25	1.11 × 10^−16^
Signal Transduction	102	4.44 × 10^−16^
Intracellular signaling by second messengers	32	1.22 × 10^−15^
Disease	62	1.78 × 10^−15^
PIP3 activates AKT signaling	30	3.33 × 10^−15^
Negative regulation of the PI3K/AKT network	21	6.11 × 10^−15^
Signaling by FGFR in disease	17	4.52 × 10^−14^
Signaling by SCF-KIT	14	1.62 × 10^−13^
**Pathway (Decreased)**	**Count**	***p*** **Value**
Neutrophil degranulation	64	1.11 × 10^−16^
Platelet degranulation	30	1.11 × 10^−16^
Regulation of IGF transport and uptake by IGFBPs	31	1.11 × 10^−16^
Innate immune system	100	1.11 × 10^−16^
Response to elevated platelet cytosolic Ca^2+^	30	2.22 × 10^−16^
Post-translational protein phosphorylation	27	4.44 × 10^−16^
Homeostasis	63	1.67 × 10^−12^
Platelet activation, signaling and aggregation	36	2.70 × 10^−13^
Immune system	121	4.97 × 10^−10^
Binding and Uptake of Ligands by Scavenger Receptor	21	2.21 × 10^−9^
